# Comparison of cooling methods to induce and maintain normo- and hypothermia in intensive care unit patients: a prospective intervention study

**DOI:** 10.1186/cc6104

**Published:** 2007-08-24

**Authors:** Cornelia W Hoedemaekers, Mustapha Ezzahti, Aico Gerritsen, Johannes G van der Hoeven

**Affiliations:** 1 Department of Intensive Care, Radboud University Nijmegen Medical Centre, Nijmegen, The Netherlands

## Abstract

**Background:**

Temperature management is used with increased frequency as a tool to mitigate neurological injury. Although frequently used, little is known about the optimal cooling methods for inducing and maintaining controlled normo- and hypothermia in the intensive care unit (ICU). In this study we compared the efficacy of several commercially available cooling devices for temperature management in ICU patients with various types of neurological injury.

**Methods:**

Fifty adult ICU patients with an indication for controlled mild hypothermia or strict normothermia were prospectively enrolled. Ten patients in each group were assigned in consecutive order to conventional cooling (that is, rapid infusion of 30 ml/kg cold fluids, ice and/or coldpacks), cooling with water circulating blankets, air circulating blankets, water circulating gel-coated pads and an intravascular heat exchange system. In all patients the speed of cooling (expressed as°C/h) was measured. After the target temperature was reached, we measured the percentage of time the patient's temperature was 0.2°C below or above the target range. Rates of temperature decline over time were analyzed with one-way analysis of variance. Differences between groups were analyzed with one-way analysis of variance, with Bonferroni correction for multiple comparisons. A *p *< 0.05 was considered statistically significant.

**Results:**

Temperature decline was significantly higher with the water-circulating blankets (1.33 ± 0.63°C/h), gel-pads (1.04 ± 0.14°C/h) and intravascular cooling (1.46 ± 0.42°C/h) compared to conventional cooling (0.31 ± 0.23°C/h) and the air-circulating blankets (0.18 ± 0.2°C/h) *(p *< 0.01). After the target temperature was reached, the intravascular cooling device was 11.2 ± 18.7% of the time out of range, which was significantly less compared to all other methods.

**Conclusion:**

Cooling with water-circulating blankets, gel-pads and intravascular cooling is more efficient compared to conventional cooling and air-circulating blankets. The intravascular cooling system is most reliable to maintain a stable temperature.

## Introduction

Temperature management is used with increasing frequency as a tool to mitigate neurological injury. Mild hypothermia has a beneficial effect on outcome in patients after out of hospital cardiac arrest [1-3]. Hypothermia also effectively lowers intracranial pressure in patients after traumatic brain injury [4-6] and was found to lower mortality in subgroups of patients [7]. In a Cochrane analysis, however, no overall benefit in terms of lower morbidity or mortality could be determined [8].

Fever is extremely common in brain-injured patients. The risk increases with the length of ICU stay from 16% for patients admitted to a neurological intensive care unit (ICU) for less than 24 hours to 93% for those staying longer than 14 days [9]. Hyperthermia exacerbates ischemic neuronal injury in patients at risk of secondary brain damage [10].

Temperature reduction is neither easy nor without risk. Induction of hypothermia can result in decreased cardiac output, arrhythmias, bleeding diathesis, electrolyte disorders and increased insulin resistance [11]. To be applicable in a larger number of patients, cooling has to be accomplished in an easy, controllable, minimally invasive and well-tolerated way. Little is known about the optimal method of temperature control. Most studies have compared a single cooling technique with medical treatment or another cooling device. The aim of this study is to compare five different cooling techniques during induction and maintaining of mild hypo- and normothermia in terms of efficiency and cooling performance.

## Materials and methods

### Study population

A total of 50 consecutive adult patients with an indication for controlled mild hypothermia or strict normothermia were prospectively enrolled. The local Institutional Review Board waived the need for informed consent. The target temperature in the mild hypothermia group was a rectal temperature of 33°C, and in the strict normothermia group the target temperature was a rectal temperature of 37°C.

The study was conducted in the ICU of a tertiary university hospital. Patients were eligible for induction of normothermia if they developed a temperature of >38.5°C for at least 30 minutes. The ICU medical staff identified the patients that required cooling to hypo- or normothermia.

Patients were excluded from the study if they had a rectal temperature <34.5°C (in the hypothermia group) or <38.5°C (in the normothermia group) at the beginning of the study. In addition, patients were excluded if they suffered from severe hemodynamic instability, severe sepsis, or active bleeding or if they received renal replacement therapy. Severe hemodynamic instability was defined as the need for increasing amounts of vasoactive support, or requiring >0.5 μg/kg/minute (nor)epinephrine. Severe sepsis was defined as sepsis with organ dysfunction/failure. Active bleeding was defined as blood loss requiring more than 2 units of erythrocyte concentrates/24 hours.

### Study intervention

Ten patients in each group were prospectively assigned to conventional cooling, cooling with a water circulating external cooling device (Blanketrol II, Cincinatti Subzero, The Surgical Company, Amersfoort, The Netherlands), an air circulating external cooling device (Caircooler CC1000, Medeco, Oud-Beijerland, The Netherlands), a water circulating external cooling device using self-adhesive gel-coated pads (Arctic Sun, Medivance, Jugenheim, Germany) or an intravascular heat exchange system (Icy-catheter, Alsius Coolgard 3000, Medicor, Nieuwegein, The Netherlands). Randomization was done by assignment of the patients in consecutive order to the different devices. Following identification by the medical staff, the patients were included in the study and allocated to a cooling method. The order of the cooling devices was determined randomly and not influenced by the clinicians responsible for the individual patients. During the test period of a specific device, no patient was cooled using any other device, unless the number of patients in need of temperature management exceeded the number of available cooling machines. In that case the additional patients were cooled using conventional cooling (considered standard cooling in our hospital) and not included in this study. In each group, five patients were cooled to hypothermia and five patients to normothermia.

Conventional cooling consisted of rapid infusion of 30 ml/kg ideal bodyweight of lactated Ringer's solution at 4°C, followed by surface cooling using ice and/or coldpacks. The timing and amount of ice and coldpacks were judged by the attending nurse and guided by the patient's temperature.

The water circulating cooling system consists of two water-circulating cooling blankets, placed under and over the patient, and a third smaller blanket under the patient's head. The large blankets have of 1.1 m^2 ^each, the smaller blanket a surface area of 0.15 m^2^, and all are connected to an automatic temperature control module guided by the rectal temperature of the patient. The temperature of the water circulating through the blankets ranges between 4°C and 42°C.

The air-circulating cooling system uses a single blanket placed over the patient with a total surface area of 1.9 m^2^. According to the manufacturer's manual, air temperature reaching the patient is within 2°C of the listed temperatures, with an airflow of 28–32 cfm. This blanket cannot be connected to an automatically guided temperature module, and was set manually at the lowest temperature possible (that is, 10°C). After the target temperature was reached, the temperature of the device was manually adjusted by the attending nurse (range 10°C to 42°C).

The gel-coated external cooling device consists of four water circulating gel coated energy transfer pads, and is placed on the patient's back, abdomen, and both thighs. Depending on the size used, the total surface area ranges between 0.60 and 0.77 m^2^. It is connected to an automatic thermostat controlling the temperature of the circulating water (range 4°C to 42°C) based on the patient's rectal temperature.

The intravascular cooling system uses a single lumen (8.5 Fr, 38 cm) central venous catheter inserted into the inferior vena cava via the left or right femoral vein. Normal saline is pumped through three balloons mounted on the catheter and returned to a central system in a closed loop. The saline flow within the balloons is in close contact with the patient's blood flow and serves as a heat exchange system. An automatic temperature control device adjusts the temperature of the circulating saline (range 4°C to 42°C) based on the patient's rectal temperature.

Conventional cooling was the standard method of temperature control in the ICU. After extensive instruction by the manufacturer, no learning curve was required for the different cooling devices. All these cooling devices were used as advised by the operator's manual and the distributor. None of the commercially available systems were pre-cooled before use. Temperature recording to measure cooling rate was started when the cooling device was connected to the patient and ready for use. In the conventional group, time was started at the start of the infusion of cold fluids. If the target temperature was not reached within 12 hours after start of the cooling, ice and cold packs were used for additional cooling. No alternative cooling was used in the patients allocated to conventional cooling.

### Standard care

All patients were admitted to the ICU, monitored and treated according to international standards. All patients were intubated and mechanically ventilated. If necessary, patients were sedated using midazolam and/or propofol to a Ramsay score of 6 and received adequate analgesia with morphine or fentanyl. If patients exhibited clinical signs of shivering they were treated with extra sedation, morphine or rocuronium as a nondepolarizing neuromuscular blocking agent. Use of paracetamol was not dictated by protocol, but left to the discretion of the attending medical staff. Vasoactive or inotropic support, usually norepinephrine or dobutamine was administered if necessary.

### Data collection

Demographic, clinical, laboratory and pharmacological data were obtained through review of the medical records of the patients. Body temperature was measured continuously using a rectal temperature probe (YSI Incorporated 401, Van de Putte Medical, Nieuwegein, The Netherlands) and recorded every 15 minutes for at least 24 hours. If the cooling device was equipped with a temperature control module, the patients received two separate rectal temperature probes, one connected to the central ICU monitoring system, the other connected to the control module of the cooling device.

The primary endpoints of the study were the initial rate of temperature decrease, expressed as °C/h and the percentage of time the temperature was out of range during the first 24 hours of treatment (defined as more than 0.2°C above or below target temperature). When the temperature was out of range, the mean temperature change from target was calculated. If the target temperature was not reached within 24 hours, treatment was considered as a failure.

Secondary endpoints of the study included occurrence of overshoot cooling (defined as a temperature drop >0.5°C below target temperature), incidence of hypotension (defined as mean arterial pressure <60 mmHg) or arrhythmia, development of skin lesions, and malfunction of the cooling device. Infections were diagnosed using CDC criteria.

### Statistical analysis

Power calculation was based on previous tests using the water-circulating cooling device and conventional cooling with ice and coldpacks. We considered a 20% difference in cooling rate as clinically important. With an estimated standard deviation (SD) of 15% and a significance level α of 0.05, a sample size of 5 patients per group was calculated to reach a power of 90%. We therefore included ten patients per group in the present study (five patients in the hypothermia group and five in the normothermia group). Rates of temperature decline over time were analyzed with one-way analysis of variance. Differences between groups were analyzed with one-way analysis of variance, with Bonferroni correction for multiple comparisons or by Chi square test as appropriate. A *p *< 0.05 was considered statistically significant. All data are expressed as mean ± SD unless otherwise stated.

## Results

### Baseline characteristics

A total of 50 patients were enrolled in the study. The clinical and demographic characteristics of the patients at randomization are shown in Table 1. No differences were found with respect to age, body mass index, or APACHE II scores. The majority of the patients treated with mild hypothermia were patients after out-of-hospital arrest with a presumed cardiac origin (Table 1). Other indications for hypothermia included in-hospital-arrest, and uncontrollable intracranial pressure after traumatic brain injury. The majority of the patients enrolled in the normothermia group had subarachnoid hemorrhage or traumatic brain injury (Table 1). Fever was most frequently of infectious origin with pneumonia as the most frequent identified cause.

**Table 1:** Baseline characteristics of patients in the hypothermia and normothermia groups

	Conventional	BR	CC	AS	CG	*P *value
**Hypothermia**						
Gender (male)	5	4	3	3	4	0.546
Age (years)	69.4 ± 16.3	64.6 ± 7.8	63.4 ± 17.6	58.8 ± 14.7	60.4 ± 14.6	0.706
APACHE II	26.8 ± 4.8	29.2 ± 5.2	22.4 ± 9.5	22.0 ± 11.8	26.2 ± 9.3	0.268
BMI (kg/m^2^)	26.3 ± 3.4	25.2 ± 1.5	26.0 ± 4.2	26.4 ± 3.8	24.4 ± 3.2	0.137
Diagnosis						
OHA	2	4	3	5	2	
IHA	2	0	0	0	2	
Cardiac origin	2	3	3	3	2	
High ICP	1	1	2	0	1	
Alive at discharge from ICU	4	1	2	4	0	0.034
						
**Normothermia**						
Gender (male)	3	4	4	3	5	0.546
Age (years)	46.4 ± 7.3	37.8 ± 14.7	49.0 ± 15.4	57.6 ± 16.2	48.8 ± 12.8	0.05
APACHE II	20.6 ± 7.9	15.6 ± 8.6	21.2 ± 9.6	24.2 ± 4.1	24.0 ± 7.3	0.770
BMI (kg/m^2^)	25.5 ± 0.5	25.9 ± 4.7	25.3 ± 2.8	25.7 ± 1.7	24.9 ± 1.8	0.868
Diagnosis						
SAH	2	0	2	3	1	
TBI	2	5	1	1	2	
Post-anoxic	0	0	2	0	2	
Intracerebral hemorrhage	1	0	0	1	0	
Cause of fever						
Pneumonia	3	3	3	5	2	
Meningitis	1	1	0	0	0	
CVC related bacteriemia	0	1	0	0	2	
SIRS	1	0	2	0	1	
Alive at discharge from ICU	5	3	0	4	5	0.003

Conventional, conventional cooling with ice cold fluids and ice/coldpacks; BR, water-circulating cooling system; CC, air-circulating cooling system; AS, gel-coated cooling system; CG, intravascular cooling system. BMI, body mass index; CVC, central venous catheter; ICP, intracranial pressure; ICU, intensive care unit; IHA, in-hospital arrest; OHA, out-of hospital arrest; SAH, sub-arachnoidal hemorrhage; SIRS, systemic inflammatory response syndrome; TBI, traumatic brain injury.

### Induction of hypo- and normothermia

**Figure 1:**
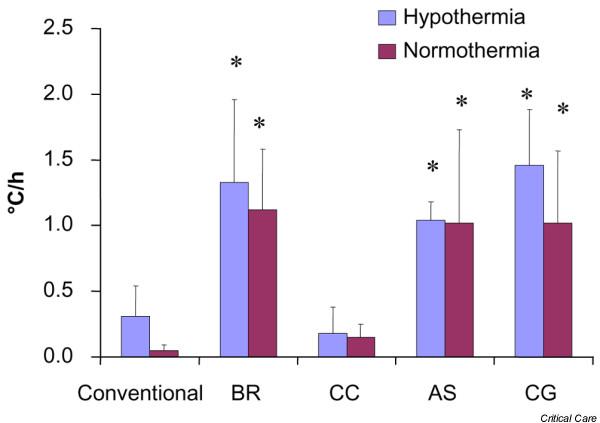
Induction of hypo- and normothermia. The pace of cooling (expressed as°C/h) in the hypothermia and normothermia groups. Bars represent mean values ± standard deviation. Asterisks indicate significant differences. Conventional, conventional cooling with ice cold fluids and ice/coldpacks; BR, water-circulating cooling system; CC, air-circulating cooling system; AS, gel-coated cooling system; CG, intravascular cooling system.

In the hypothermia group, the speed of cooling (expressed as °C/h) was significantly higher in the patients cooled with the water-circulating cooling device (1.33 ± 0.63°C/h), the gel-coated external device (1.04 ± 0.14°C/h) and the intravascular catheter (1.46 ± 0.42°C/h) compared to both the air-circulating cooling device (0.18 ± 0.20°C/h) and conventional cooling (0.32 ± 0.24°C/h) (*p *< 0.05) (Figure 1). Similar results were found in the normothermia group, with a mean temperature decrease of 1.12 ± 0.46°C/h in patients cooled with the water-circulating cooling device, 1.02 ± 0.71°C/h with the gel-coated device and 1.02 ± 0.55°C/h with the intravascular catheter compared to both 0.15 ± 0.10°C/h with the air-circulating cooling device and 0.06 ± 0.05°C/h with conventional cooling (*p *< 0.05; Figure 1).

Additional cooling with ice and cold packs was necessary in two patients in both the hypothermia and normothermia groups cooled with the air-circulating cooling device (Table 2). Treatment failure, defined as failure to reach the target temperature within 24 hours after start of cooling, occurred in 2 hypothermia patients with conventional cooling, 2 hypothermia patients cooled with the air-circulating device, 4 normothermia patients with conventional cooling and 1 normothermia patient cooled with the air-circulating device. Use of sedatives and analgesics differed (non-statistically) between groups (Table 2). Five patients were treated without the use of sedation. These patients were comatose after cardiac arrest with a Glasgow Coma Score of 3 and showed no signs of discomfort or shivering while cooling to hypothermia (two patients) or normothermia (three patients). In the hypothermia group, neuromuscular blocking was necessary in two patients with conventional cooling, three patients cooled with the air-circulating and water-circulating systems, five patients cooled with the gel-coated cooling device and five patients cooled with the intravascular cooling system. In the normothermia group, neuromuscular blocking was used in no patients with conventional cooling, three patients cooled with the air-circulating and water-circulating systems, four patients cooled with the gel-coated cooling device and five patients cooled with the intravascular cooling system.

**Table 2:** Patient characteristics during cooling to hypo- and normothermia in the hypothermia and normothermia groups

	Conventional	BR	CC	AS	CG	*P *value
**Hypothermia**						
Sedatives	5	5	3	5	5	0.069
Neuromuscular blockers	2	3	3	5	5	0.129
Analgesics	5	4	3	5	5	0.195
Paracetamol	3	0	3	2	2	0.287
Inotropic agents	5	3	3	2	4	0.311
Vasodilatory agents^a^	0	1	3	0	1	0.112
Adverse events						0.271
Hypotension^b^	2	2	1	2	2	
Arrhythmia^c^	0	2	2	3	2	
Skin lesions	0	0	0	0	0	
Overshoot^d^						0.058
No. of patients	1	3	0	3	0	
Lowest temperature (°C)	31.9	31.0 ± 0.3		32.4 ± 0.1		
Use of additional cooling	0	0	2	0	0	0.069
Treatment failure^e^	2	0	2	0	0	0.129
						
**Normothermia**						
Sedatives	4	3	5	5	5	0.195
Neuromuscular blockers	0	3	3	4	5	0.195
Analgesics	4	3	5	5	5	0.195
Paracetamol	5	5	4	4	5	0.515
Inotropic agents	1	0	2	3	2	0.311
Vasodilatory agents^a^	0	0	1	0	0	0.384
Antibiotics	4	5	5	5	5	0.384
Adverse events						0.069
Hypotension^b^	0	0	1	0	0	
Arrhythmia^c^	0	0	1	0	0	
Skin lesions	0	0	0	0	0	
Overshoot^d^						0.040
No. of patients	0	3	0	2	0	
Lowest temperature (°C)		35.7 ± 0.4		36.1 ± 0.1		
Use of additional cooling	0	0	2	0	0	0.069
Treatment failure^e^	4	0	1	0	0	0.019

Conventional, conventional cooling with ice cold fluids and ice/coldpacks; BR, water-circulating cooling system; CC, air-circulating cooling system; AS, gel-coated cooling system; CG, intravascular cooling system. ^a^Vasodilatation used low dose nitroglycerin or ketanserin iv. ^b^Hypotension is defined as mean arterial pressure ≤ 60 mmHg. ^c^Arrhythmia defined as any rhythm but normal sinus rhythm, sinus bradycardia or sinus tachycardia. ^d^Overshoot defined as drop of body temperature during initiation of cooling >0.5°C below target temperature. ^e^Treatment failure defined as failure to reach target temperature within 24 hours after start of cooling.

### Maintaining hypo- and normothermia

**Figure 2:**
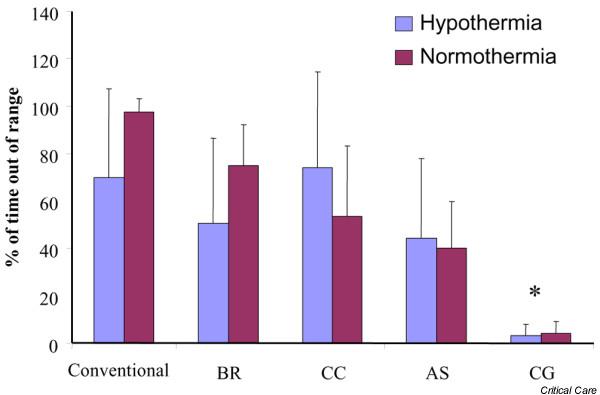
Maintaining target temperature. The ability of the cooling device to maintain a stable target temperature is depicted as the percentage of time the patient's temperature was 0.2°C below or above the target temperature. Bars represent mean values ± standard deviation. Asterisks indicate significant differences. Conventional, conventional cooling with ice cold fluids and ice/coldpacks; BR, water-circulating cooling system; CC, air-circulating cooling system; AS, gel-coated cooling system; CG, intravascular cooling system.

After the target temperature was reached, we measured the percentage of time the patient's temperature was 0.2°C below or above the target temperature. Compared to all other cooling methods, the intravascular cooling device was significantly more reliable in keeping the patients within the target range (Figure 2). In the hypothermia group the intravascular catheter was 3.2 ± 4.8% of the time out of range compared to 69.8 ± 37.6% with conventional cooling, 50.5 ± 35.9 with the water-circulating cooling device, 74.1 ± 40.5% with the air-circulating cooling device and 44.2 ± 33.7% with the gel-coated external cooling system (*p *< 0.05). Similar results were found in the normothermia group: the intravascular catheter was 4.2 ± 5.1% of the time out of range compared to 97.4 ± 5.8% with conventional cooling, 74.8 ± 17.4 with the water-circulating cooling device, 53.6 ± 29.5% with the air-circulating cooling device and 40.2 ± 19.5% with the gel-coated external cooling system (*p *< 0.05).

**Figure 3:**
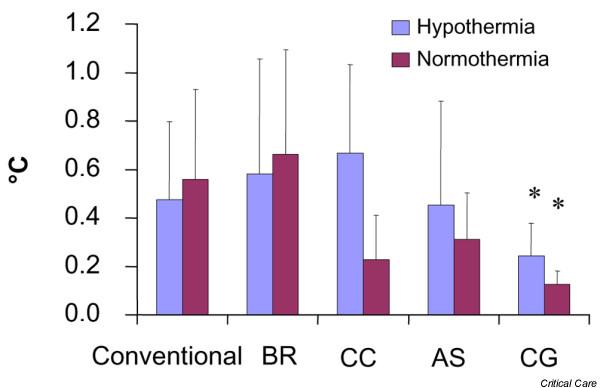
Temperature deviation from target temperature. Mean temperature deviation after induction of hypothermia or normothermia while maintaining the target temperature. Bars represent mean values ± standard deviation. Asterisks indicate significant differences. Conventional, conventional cooling with ice cold fluids and ice/coldpacks; BR, water-circulating cooling system; CC, air-circulating cooling system; AS, gel-coated cooling system; CG, intravascular cooling system.

Mean temperature deviation from the target temperature in the hypothermia group was significantly lower in the patients cooled with the intravascular catheter (0.24 ± 0.14°C) compared to all other groups: conventional cooling (0.48 ± 0.3°C), the water-circulating cooling device (0.58 ± 0.47°C), the air-circulating cooling device (0.67 ± 0.36°C), and the gel-coated external cooling system (0.45 ± 0.42°C) (Figure 3) (*p *< 0.05). Mean temperature deviation from the target temperature in the normothermia group was significantly lower in patients cooled with the intravascular catheter (0.13 ± 0.06°C) compared to conventional cooling (0.56 ± 0.38°C), the water-circulating cooling device (0.66 ± 0.43°C), the air-circulating cooling device (0.23 ± 0.18°C), and the gel-coated external cooling system (0.31 ± 0.19°C) (Figure 3) (*p *< 0.05).

### Adverse events

In the hypothermia group, a drop of body temperature during initiation of cooling of more than 0.5°C below the target temperature was found in 1 patient with conventional cooling, 3 patients cooled with the water-circulating cooling device and 3 patients with the gel-coated external cooling device. In the normothermia group, overshoot was found in three patients cooled with the water-circulating cooling device and two patients with the gel-coated external cooling device.

Hypotension and arrhythmia were observed only in hypothermia patients without differences between the groups (Table 2). This occurred exclusively in patients after cardiac arrest and may have resulted from the underlying condition rather than a specific cooling method. The use of inotropic agents was comparable between the groups. Hypotension or use of inotropic support was not related to speed of cooling or occurrence of overshoot cooling. Malfunctioning of a cooling device did not occur. Skin lesions or catheter-related events, such as thrombosis or infection, were not reported.

## Discussion

This is the first study comparing the efficiency and safety of five different cooling methods in inducing and maintaining hypo- and normothermia in ICU patients. Cooling using water-circulating blankets, gel-coated water circulating pads and intravascular cooling was equally efficient in inducing hypo- and normothermia. Intravascular cooling was superior to all other cooling methods for maintaining a stable target temperature. No adverse events related to a specific cooling method were documented. The absence of adverse events should, however, be interpreted with caution because of low numbers.

In our trial, induction of cooling using water-circulating blankets, water-circulating gel pads or intravascular cooling was equally effective. A previous comparison between water-circulating blankets and gel pads in febrile ICU patients found that cooling with gel pads was significantly more effective than blankets in reducing fever [12]. This may be explained by the fact that, in that trial, a single water blanket was used with a surface area of only 0.92 m^2^. We used three water-circulating cooling blankets with a total surface area of 2.35 m^2^. The rate of cooling with the gel-pads in our trial is comparable with results from previous trials [13,14], indicating that the performance of this cooling device was similar in our patients. Intravascular cooling was equally effective in inducing the target temperature compared to water blankets and gel pads. Previously, intravascular cooling has been shown to be more effective than air- and water-circulating blankets in both inducing and maintaining hypothermia [15]. External cooling was significantly less efficient in our trial, possibly explaining the superiority of the endovascular catheter in this study. The superiority of endovascular cooling is most likely due to the direct heat-exchange between catheter and blood, resulting in a rapid transfer of cold blood through the body, whereas surface cooling depends on relatively slow conduction of cold mainly through the tissue itself. The effectiveness of devices with an automatic temperature control module was higher compared to manually operated methods. It is unlikely, however, that control of temperature fully accounts for the lack of efficiency. At the initiation of cooling all devices were set to their maximum performance, yet the speed of cooling in the induction phase was lower in the manually operated methods. In the case of slow or inadequate regulation by the nursing staff, we would have expected cases of severe hypothermia, which was not the case in this series.

In terms of labour, the methods without an automatic temperature feedback module required constant supervision by the nursing staff and were most labour intensive. The endovascular method required the insertion of a central venous line; this drawback is relative since most patients in the ICU need central venous access under these conditions. The cost of the different devices is mainly determined by the use of the disposables. The endovascular cooling system was most expensive (approximately 1,000 Euro per patient) followed by the gel coated surface cooling (approximately 700 Euro per patient), the air circulating device (approximately 25 Euro per patient) and the water circulating blanket (approximately 25 Euro per patient).

Conventional cooling was not effective in our study and resulted in treatment failure in 60% of our patients. This is in contrast with other studies showing an average temperature decrease of 1.7°C to 2.5°C per hour [16-18]. An even higher temperature decrease of 4°C in the first hour was found by Polderman and colleagues [19], who combined ice-cold fluids with a water-circulating cooling device. In our trial, conventional cooling was induced by rapid infusion of 30 ml/kg ideal bodyweight of lactated Ringer's solution at 4°C. The speed of infusion was not dictated by protocol whereas in the study by Polderman and colleagues, 1,500 ml of fluid was infused in 30 (no cardiac shock) or 60 minutes (cardiac shock). In addition, Polderman and colleagues used water circulating blankets in addition to the infusion of cold fluids. Application of ice or coldpacks may have been less efficient compared to this cooling device. The lack of effectiveness in our study may be the result of slower infusion rates, lower volumes, or inadequate amounts of ice and coldpacks.

Cooling was less efficient in normothermia compared to hypothermia. At normothermia the body's control mechanisms to maintain the centrally mandated target temperature are working at maximum efficiency. In addition, in hyperthermic patients, the central thermostat may be influenced by inflammation, or be deregulated by primary neurological damage. In hypothermia the body's re-warming mechanisms are less effective, especially when the body temperature drops below 33°C.

There are several limitations to this study. The nursing staff and attending doctors could not be blinded to treatment allocation for obvious practical reasons. It is unlikely that this would have influenced the outcomes of this study since the cooling devices were operated strictly according to the operators' manuals, and temperatures were recorded automatically.

The use of sedatives, analgesics and neuromuscular blocking agents differed between the groups. These drugs were administered only in case of shivering and distress, and their prescription was left to the discretion of the attending medical staff not involved in this clinical trial. In humans, core temperature is normally maintained within a tight range. A reference temperature (set point) generated by a network of warm, cold, and thermal insensitive neurons in the pre-optic area is compared with feedback from the skin and core thermoreceptors. An error signal, proportional to the difference between the set point and feedback signal, is generated, which activates thermoeffector pathways, including vasoconstriction and shivering. A larger difference between set point and feedback signal will thus result in more intense vasoconstriction and shivering. This was also the case in our trial: the devices that resulted in a stronger decrease of the feedback signal induced shivering more frequently. In this study, patients were sedated to a Ramsay score of 6 and received adequate analgesia with morphine or fentanyl. If patients exhibited clinical signs of shivering, they were treated with extra sedation, morphine or muscle relaxation. In our ICU, this is the normal protocol in patients that need temperature management. Most studies that compare different cooling devices use a similar protocol of sedation and relaxation [19-24]. In those studies as well as in our study, patients treated with the most efficient cooling device needed more sedation and relaxation. Since this was caused by the stronger temperature decline in these patients, differences in use of sedation and relaxation is considered a consequence rather than cause of efficient cooling.

Pulmonary artery core temperature is considered the gold standard for measurement of core body temperature [25-28]. A major disadvantage is the invasive nature of this technique and its relatively high cost. Rectal temperature is comparable to pulmonary artery core temperature (mean difference of 0.07 ± 0.4°C) and has a time lag of approximately 15 minutes [29]. This technique was chosen because it is common practice in most ICUs. In addition, the water-circulating cooling device, the gel-coated external cooling system and the endovascular cooling system are all equipped with an automatic temperature control device based on the patient's rectal temperature. Previous studies comparing different devices also used non-invasive temperature measurement. To ensure that the results of this study are applicable to most ICUs and comparable to previous studies, we chose to measure temperature in a non-invasive way.

## Conclusion

The results of our study demonstrate that water-circulating blankets, gel-coated water circulating pads and intravascular cooling are equally efficient in inducing hypothermia and normothermia. For maintaining the target temperature, intravascular cooling is superior to all other cooling methods.

## Key messages

• Cooling with water-circulating blankets, gel-pads and intravascular cooling is more efficient compared to conventional cooling and air-circulating blankets.

• The intravascular cooling system is most reliable to maintain a stable temperature.

• No adverse events related to a specific cooling method were documented.

## Abbreviations

ICU = intensive care unit; SD = standard deviation.

## Competing interests

The authors declare that they have no competing interests.

## Authors' contributions

All authors participated in the design and coordination of the study and draft of the manuscript. All authors read and approved the final manuscript.
